# Governance Choices of Genome Editing Patents

**DOI:** 10.3389/fpos.2021.745898

**Published:** 2021-09-06

**Authors:** Naomi Scheinerman, Jacob S. Sherkow

**Affiliations:** 1 Department of Medical Ethics and Health Policy, Perelman School of Medicine, University of Pennsylvania, Philadelphia, PA, United States; 2 College of Law, University of Illinois at Urbana-Champaign, Champaign, IL, United States; 3 Carl R. Woese Institute for Genomic Biology, University of Illinois at Urbana-Champaign, Urbana, IL, United States; 4 Centre for Advanced Studies in Biomedical Innovation Law, Faculty of Law, University of Copenhagen, Copenhagen, Denmark

**Keywords:** CRISPR, patent, democracy, governance, law

## Abstract

There are a variety of governance mechanisms concerning the ownership and use of patents. These include government licenses, compulsory licenses, march-in rights for inventions created with federal funding, government use rights, enforcement restrictions, subject-matter restrictions, and a host of private governance regimes. Each has been discussed in various contexts by scholars and policymakers and some, in some degree, have been employed in different cases at different times. But scholars have yet to explore how each of these choices are subject to—or removed from—democratic control. Assessing the range of democratic implications of these patent governance choices is important in understanding the social and political implications of controversial or wide-ranging technologies because their use has a significant potential to affect the polity. This paper seeks to unpack these concerns for genome editing, such as CRISPR, specifically. Patents covering genome editing make an interesting case because, to date, it appears that the polity is concerned less with certain kinds of access, and more with distribution and limits on the technology’s particular uses, such as human enhancement and certain agricultural and environmental applications. Here, we explore what it means for patents to be democratic or non-democratically governed and, in so doing, identify that patents covering many of the most controversial applications—that is, ones most likely to gain public attention—are effectively controlled by either non- or anti-democratic institutions, namely, private restrictions on licensing. This may be effective—for now—but lawmakers should be wary that such restrictions could rapidly reverse themselves. Meanwhile, other choices, like compulsory licenses, more broadly touch on democratic deliberation but, as currently structured, are aimed poorly for particular applications. Insofar as the public wants, or perhaps deserves, a say in the distribution and limits of these applications, illuminating the ways in which these governance choices intersect—or fail to intersect—with democratic institutions is critical. We offer some concluding thoughts about the nature of patents and their relationship with democratic governance as distributed claims to authority, and suggest areas for scholars and policymakers to pay close attention to as the genome editing patent landscape develops.

## INTRODUCTION

Few technologies are recognized as revolutionary immediately upon their invention. CRISPR—a form of altering DNA sequences inside living cells, or “genome editing”—stands out among those (Jinek et al., 2012; Gasiunas and Siksnys, 2013). Like other revolutionary technologies, controlling genome editing through typical channels of democracy has been a challenge and a matter of public concern (NASEM Genome Editing Report, 2017; WHO Genome Editing Report, 2021). Patents—legal instruments giving their bearers a right to exclude others from practicing a particular invention—have been proposed as governance tool (Guerrini et al., 2017; Parthasarathy, 2018), but the democratic implications of such a governance mechanism has been largely unexplored. In this paper, we examine patent licensing regimes—laws regarding the limits of how patents can be licensed to others—as a governance mechanism for CRISPR and assess these regimes’ democratic implications. While many licensing regimes rely on forms of representative democracy, they also seem amenable to broader forms of participatory democracy, the latter of which may be more effective than omnibus attempts to control a widely distributed technology. Given this, a principal democratic path to controlling genome editing lies in, of all things, patent licensing regimes.

CRISPR is a form of genome editing, the ability to alter the constituent DNA of a living cell (its “genome”), at will using an engineered—and infinitely malleable—bacterial immune system (Jinek et al., 2012; Gasiunas et al., 2012; Cong et al., 2013). It is cheap, easy, and flexible; it has worked in every type of organism yet experimented on (Gustafson, 2020). But this ease at editing the genome brings with it the potential for societally controversial applications, such as “designer babies” (Greely, 2021). Many of these are, frankly, little more fantasy, but the power of the technology has instilled both awe and fear in the greater public (Maxmen, 2015). Notably, CRISPR is subject to its own body of dystopian literature (Ishiguro, 2021), impressive, given that the technology is not even yet a decade old. And it is has been heralded by one of its inventors as the “holy grail” of molecular biology, a bold statement with few, if any, opponents (Gasiunas and Siksnys, 2013).

As a powerful, commercially valuable technology, CRISPR is subject to a broad patent estate. Foundational patents covering a basic iteration of the technology are owned by the Broad Institute in Cambridge, Massachusetts (a joint effort between MIT and Harvard University) and the University of California, Berkeley, among other collaborating academic institutions (Contreras and Sherkow, 2017). Beyond these patents, there are yet more, held by a variety of academic centers and research institutions around the globe (Egelie et al., 2016). But CRISPR technology is rapidly evolving, encompassing ever broader ways of effectuating genome editing among other applications (Porto et al., 2020; Marzec et al., 2020). The patent estate has similarly evolved (Bire et al., 2021).

While certainly not ignored—and indeed, explicitly mentioned by the WHO’s recent report on governing human genome editing (WHO Genome Editing Report, 2021)—patents have largely been overlooked as an instrument of governance of genome editing. This is a somewhat surprising aspect of technology studies scholarship because patents are—if not else—a legal instrument designed to limit the use of a given technology (Boldrin and Levine, 2005). Licenses are permissions to use a patented technology on terms set by patent holder. This means, accordingly, that patents control who can use a given technology, on what terms, where, and when (Guerrini et al.,2017). But not all licenses are mere arms-length agreements among patent holders and interested parties. They are also subject to licensing regimes at the mercy of government and restrictions—beyond simple economic ones—from private parties. On the government side of the ledger, these regimes include government licensing provisions, march-in rights, government-use rights, compulsory licenses, and licensing restrictions. In each of these, and as detailed below, the government either has an interest in the technology to practice it on its own behalf or to compel the patent holder to allow another to practice it in a way government deems fit. Beyond these, private licenses—although there is no requirement to do so—may set ethical conditions on the use of a given technology. This is currently happening for genome editing with the Broad Institute and others imposing ethical licensing restrictions on genome editing, including prohibiting licensees from engaging in some of its more controversial applications (Guerrini et al., 2017).

These licensing regimes—despite all ultimately being forms of technology control—have differing intersections with democratic theory. Some are receptive to the usual instruments of representative democracy, such as the polity’s support for research funding for certain applications and not others. Other regimes are one step removed, those where legislative representatives have petitioned patent holders to change their licensing practices. At other end these examples lies private licensing regimes, like those from the Broad Institute, that seem, at first blush, entirely undemocractic. But they are likely similarly receptive to faces of lay, participatory democracy, the populous demanding measures from private actors wielding significant amount of power.

Understanding all of this should be important to theorists and policymakers alike. For theorists, it brings patent licensing as a democratic mechanism of technology control, however successful, to the fore. It also suggests that patent licensing—long thought of as an elitist business—has more nuanced democratic implications, especially for controversial technologies like genome editing. This should similarly be useful to policymakers and advocacy groups seeking legally salient mechanisms to control technology in manners responsive to broader constituencies. This paper examines these features—patentsas technology governance, and patent licensing as democratic instrument—in two parts.

### Patents, Patent Licensing, and Technology Governance

#### Patents as Legal Instruments

Patents are one form, among many, of intellectual property. They are legal instruments that protect inventions from being copied by others without permission of the patent holder. In this way, patents operate as a right to *exclude* others from making, using, selling, or importing particular inventions (35 U.S.C. § 271(a))—not, as is commonly misunderstood, an affirmative right to use them.

This right to exclude is a limited right and only operates for a limited time: all patents expire, currently 20 years from the date when their underlying applications are filed. While international treaties harmonize a variety of the world’s patents laws—including this expiration period (TRIPS Agreement, 1994)—patents are domestic creatures only. US patents, for example, are only enforceable in the United States; UK patents only in the United Kingdom; and so on.

Moving from a patent application to a government-issued patent is nontrivial. Around the world patents undergo a substantive examination to assess whether the claimed invention is worthy of protection. Inventions sought to be patented must meet a variety of statutory requirements; in particular, that the underlying invention is new, useful, and—as the concept is articulated in US law—“nonobvious,” i.e., a significant improvement over the prior state of the art (35 U.S.C. § 103). The patent document itself must also properly disclose inventions to the world, broadly enabling those with skill in the art to make and use and invention; describing the invention fully and with particularity; and noting that the invention has some nontrivial use (35 U.S.C. § 112(a)). In addition, patents conclude with claims—single sentence recitations of the underlying invention—that define the specific contours of the patent right (35 U.S.C. § 112(b)). Claims, too, must be sufficiently specific and intelligible to those with skill in the patent’s art.

Patents are also not self-enforcing; they must be policed by their owners. This is typically accomplished through litigation, i.e., suits for patent infringement. Generally speaking, an entity infringes a patent where they use the claimed technology in some manner without the permission of the patent holder (35 U.S.C. § 271). The remedy, if there is a finding of infringement, is often either a measure of damages to compensate the patent holder (typically, a royalty) or a court-ordered injunction, stopping the infringer from the accused activity.

Despite these limitations, patents are powerful instruments. Patent infringement, while not a crime, can bring with it serious financial penalties. In the United States, damages for major patent infringement disputes now routinely eclipse $1 billion USD (Kass, 2020). Further, patents’ right to exclude may mean that two sets of overlapping patents will block others from practicing a larger invention without an agreement among all relevant patent holders—a case of “blocking patents.” Patents are, in effect, legal instruments governing the use of a particular technology (Smith, 2002).

### Patent Licensing and Technology Governance

Whatever role patents play in technology development—a hotly contested area of scholarly debate—government policies concerning patent licensing have the potential to ultimately affect access, distribution, and conditions of use on particular technologies. Patent licenses are permissions from patent holders to use a given piece of technology. Like other property rights, patent licenses are subject to a variety of government policies regarding when, whether, and to what extent they can be used. Those policies, the most significant of which are catalogued here, further bring with them important choices about democracy and polity—who, ultimately, has rights to access the technology and under which conditions.^1^ For purposes of this paper, we focus less on those decisions as effectuated through substantive patent law—that is, laws concerning which inventions get patented in the first instance, like the nonobviousness requirement—and turn our attention instead to policies surrounding patent licensing and their relationship to democratic or nondemocratic institutions of power. While we focus primarily on United States licensing practices, we note that many of the licensing policies described here have close analogues around the globe—or, in other cases, are harmonized by treaty. Licensing restrictions, potentially more than substantive patent law, have potential to speed or hinder technological development, to place it in the hands of a select few or many, and to decide which applications can be broadly used and under what conditions. Government policies on technology licensing are, too, policies of technological governance and have implications for democratic oversight.

## GOVERNMENT LICENSES

Through extramural grants and other programs, governments often fund a substantial amount of research and development within their borders—globally, about $2 trillion USD per year (Sargent, 2020). In the United States, the Bayh-Dole Act allows, but does not require, recipients of certain types of government funding to patent inventions created under their stead. In doing so, however, funding recipients must agree to grant to the United States, a “nonexclusive, nontransferable, irrevocable, paid-up license to practice or have practiced for or on behalf of the United States any subject invention throughout the world” (35 U.S.C. § 202(c) (4)). To be clear, this license applies only to the United States government; the patent holder is free to license—or refuse to license—the patented invention to others. But the Bayh-Dole Act’s government license provisions mean, in essence, that the government funding agency can use the patented invention for free. Many countries have similar laws.

Taken broadly, this regime—that public funding grants the government the right to freely use a patented invention—can be construed as a mixed public-private governance mechanism for the development and use of technologies. At the outset, governments must choose which technologies to fund, decisions that are ideally responsive to the desires of the underlying polity. Cancer research, for example, receives a substantial amount of government funding because combatting cancer is politically popular (Best, 2012). Picking and choosing which technologies to further develop is then, often, left to groups of independent experts charged with choosing the projects most likely to be successful (Price, 2019). The ultimate technology developed—if anything—is then a product of the funding recipient’s own efforts (and, of course, chance). And it is the funding recipient, not the government, that gets to choose whether to patent any resulting inventions or place them in the public domain. In this way, the Bayh-Dole Act its government license provisions control how publicly funded technology is both created and, to a certain degree, disseminated back to the government if not the public writ large.

### March-in Rights

Related to the Bayh-Dole Act’s government license provisions are the Act’s rules regarding “march-in rights.” March-in rights allow a government funding agency to “march in” and forcibly grant others a patent license for a funded technology if the funding recipient has not sufficiently commercialized the invention. As set forth in the relevant statute, march-in can occur if the patented invention has not “achieve[d] practical application” or is needed to “alleviate health or safety needs,” among other cases (35 U.S.C. § 203(a)). Notably, while threats of exercising march-in rights occur from to time, no U.S. agency has ever formally used the provision (O’Brien, 2013; Thomas, 2016).

Like government licenses, march-in rights, too, can be viewed as a mixed public-private governance mechanism for the distribution of technologies. Again, the public chooses which broader area of technology to fund, while private funding recipients largely direct which implementation of that technology gets developed and whether it will be patented. The benefit of this bargain, in theory, is that the public will have practical, commercial access to the technology, once developed. But where the funding recipient or later patent holder has not commercialized the underlying technology to make it available to the public, government (and, presumably, the polity) has authority to wrest it from private hands. In theory, at least, such governance is a balance between private rights and public benefits from the technology it, itself, has funded. And indeed, recent march-in threats have been couched in just such terms. A 2016 march-in petition directed to the National Institutes of Health and signed by 51 members of Congress requested the agency use its march-in authority “to respond to the soaring cost of pharmaceuticals” by licensing patents “developed with taxpayer funds, [that] are keeping those in need from being able to access care” (Doggett, 2016). March-in, is consequently, a governance mechanism over government funded research “intended to distribute the fruits of those labors to the public” (Thomas, 2016).

## GOVERNMENT USE RIGHTS

Apart from licensing those inventions which it funds, the government also possesses the right to use inventions owned and patented by private entities. In the United States, one statute, 28 U.S.C. § 1498(a), allows the government to use or manufacture a patented invention “without license of the owner thereof.” Unlike government licenses or march-in rights, however, such a use is not free: the government, after a trial, must pay the patent owner a “reasonable and entire compensation for such use and manufacture.” (28 U.S.C. § 1498(a)). This provision, colloquially referred to as § 1498, has, in fact, been used in the United States in the past, most notably, in the late 1950s when the Military Medical Supply Agency used the provision to cut costs on tetracycline, a popular antibiotic, for personnel (Silverman and Lee, 1974). More recently, government threats of using § 1498, have encouraged recalcitrant patent holders to either cut costs in supplying their wares to the government (as with ciproflaxin, the antibiotic used following the 2001 anthrax scare) or enter into other arrangements (as with sofosbuvir, the hepatitis C drug) (Brennan et al., 2016).

Section 1498, consequently, can also be viewed as an instrument of technology governance, a public restriction on private ownership of patented technology. It essentially removes the right of patent holders to forbid the government from using the claimed technology, irrespective of the technology’s development history or its genesis from the coffers of government. The public—faced with outsized expenses regarding a particular technology or some other pressing need—can move patented technology from behind private walls into the public sphere, so long as the government pays the patent owner compensation for its use. This larger conflict regarding access to privately owned technology sounds in various aspects of democratic theory concerning the public’s right to safety, health, and welfare and its power to use purely private property to effectuate such ends (Smith, 2002).

## COMPULSORY LICENSES

Analogous to § 1498, are compulsory patent licenses, the requirement a private patent holder license the claimed technology to another private entity. While compulsory licenses are essentially unheard of in American law, they are well defined by international treaty, such as the 1994 TRIPS Agreement. Compulsory patent licenses have recently been used in Brazil, Ecuador, India, South Africa, and Thailand, among other countries (Thomas, 2014; Resolutión No, 2021. LO-001-2021). These licenses have been principally granted to generic drug manufacturers for the purpose of lowering drug costs. In addition, the compulsory license regime contemplated by the TRIPS Agreement has been the subject of some current controversy with respect to patents covering COVID-19 vaccines. The governments of India and South Africa, in particular, have argued that the Agreement’s compulsory licensing processes are too lengthy and burdensome to engage in during the COVID-19 pandemic; they have asked the Agreement’s oversight body, the World Trade Organization, to waive these (and other) procedures (2 October Waiver Request, 2020). Other countries have followed suit (25 May Waiver Request, 2021).

Compulsory licenses, in this way, present many of the same governance choices as does § 1498, i.e., a public restriction on private ownership of patented technology. While they have largely been used in the public health context—to lower drug costs, for example—compulsory licenses occupy a broader institutional power. They can be used, under article 31 of the TRIPS Agreement, for cases of “national emergency or other circumstances of extreme urgency”—a readily pliable standard. Compulsory licenses are, therefore political choices concerning the distribution of private property in cases of extremis—when too few own too much of a beneficial good, and the government’s rights in expanding access. And, like other political choices, they are a resolution a societal tensions between the government’s role in respecting private property and democratic process concerning its distribution. One analysis of compulsory patent licenses in Canada—and their diminishment following the North American Free Trade Agreement—characterized this tension in compulsory licenses as just that: between “subjecting domestic law to corporate-led agreements…[and] democratic process in Canada” (Mohamed and Chaufan, 2020).

## LICENSING RESTRICTIONS

Whereas compulsory licenses allow the government to compel patent license to others—that is, without the permission of the patent-holder—the government also has the power to restrict patent licenses if the underlying license agreements violate public policy. There are a variety of circumstances under which such restrictions arise, although they mainly center on various aspects of promoting market competition. One particularly prevalent example concerns “reverse payment agreements,” the practice of patent-holders paying others to take licenses to their technologies, often because the underlying patents are of questionable validity or it is a cost-effective way at keeping others out of the market for a given period of time (FTC v. Actavis, Inc., 2013). There are also restrictions on licensing patents beyond their expiration date (Kimble, 2015 v. Marvel Entertainment, LLC, 2015); licensing patents to cover technology beyond that protected by the patent (Princo Corp. v. ITC, 2010); and licensing patents in a collusive manner (Illinois Tool Works, Inc. v. Independent Ink, Inc., 2006). In the United States, resolving these tensions are difficult, but active policing of licensing restrictions are minimal relative to the quantity of licensing and litigation otherwise present. In Europe, by contrast, it is an active area of public litigation despite the recognition that patent protection is in many ways, itself, anticompetitive (Petit, 2017).

Restrictions on patent licensing govern circumstances under which private agreements regarding access to technology. Unlike some of the other cases described above, these do not immediately concern the public’s use of the technology or, as with § 1498 or compulsory licensing, the government picking winners and losers to use technology. Instead, they center on platting a level (and broad) playing field for private participation in a given technology. This is layered on an already substantial literature exploring the relationship between democratic ideals and the antitrust laws. Recently, for example, Lina M. Kahn drew a deep connection between the market power of online platform services, such as Amazon, and their potential to diminish democratic values, even in absence of traditional antitrust concerns like consumer welfare (Kahn, 2017). Patent licensing restrictions, therefore, can be seen as a governance choice—sometimes imbued with democratic ideals—regarding the private development of technologies.

## PRIVATE GOVERNANCE REGIMES

Beyond these public patent licensing governance regimes, there is a wealth of private ones. Private patent holders largely possess the right to license their patents to whom they want to on, and on a variety of financial and practical terms. The largest divide, perhaps (at least in terms of access and distribution to a given patented technology) is whether the license is exclusive or nonexclusive—that is, whether the technology will be licensed exclusively to a single other entity or broadly licensed among a variety of market participants. But there are, to be sure, various gradations in between (Graff and Sherkow, 2020).

This right to license brings with it a right to establish licensing conditions governing specific uses or development restrictions over a particular technology, i.e., barring licensees from engaging in particular veins of research or development. This occurred most recently with a suite of genome editing patents owned by the Broad Institute. The Broad Institute, for its patents covering its CRISPR technologies, established a tiered regime system for its licenses concerning whether they were used for academic research, tool development, or commercial products (Contreras and Sherkow, 2017). In addition, the Broad Institute forbid its licensees from engaging in research pertaining to various controversial applications of CRISPR genome engineering, including “gene drives,” sterile seed technology, tobacco enhancement, or human germline engineering (Guerrini et al., 2017). Contrapositively, others have pledged not to enforce their patents against others, unless users were engaged in various forms of unethical behavior. This was, perhaps, most famously proposed by the scientist Kevin Esvelt, regarding using CRISPR in an inheritable, “gene drive” form (Parthasarathy, 2018).

These private license restrictions are themselves a form of private governance, here, a profit-seeking company’s autonomy to determine how a technology gets developed and on what terms. In some instances, they are commendable and dovetail with governance values centered around attention and expertise. Oftentimes, private licensors are experts in the technology’s field and know most about a given technology’s societal dangers and technical pitfalls (Guerrini et al., 2017). But such private license regimes can, in many ways, be antidemocratic. They do not, in any appreciable sense, allow the public input in what uses will and will not be restricted. These challenges notions of transparency and legitimacy in technology development. In the words of Shobita Parthasarathy, private patent license regimes “seem ill-equipped to address complex societal and value-based concerns in an increasingly privatized world” (Parthasarathy, 2018).

## CRISPR PATENTS AND TECHNOLOGY GOVERNANCE

### The CRISPR Patent Estate

Since CRISPR genome-editing technology was first described in 2012 (Jinek et al., 2012; Gasiunas et al., 2012), governments around the world have issued patents covering various forms of CRISPR-based technology. Arguably, the most famous of these are patents held separately by the Broad Institute and the University of California covering one particular iteration of CRISPR genome-editing, the use of the Cas9 enzyme to cleave a target DNA molecule and a single-guide RNA (sgRNA) to direct Cas9 to its specific, desired location (Contreras and Sherkow, 2017). Those patents have been the subject of a particular contentious patent dispute between the two institutions. In the United States, that dispute continues to rage on, and indeed has grown substantially larger since its inception in 2016. In Europe, the University of California has largely won, with the European Patent Office ruling against the Broad Institute (Zyontz and Pomeroy-Carter, 2021). But there remain persistent disputes pertaining to inventorship over this foundational iteration of the technology.

Beyond these patents, the number of CRISPR patents and patent applications has exploded since the technology’s invention. A seminal 2016 paper by Egelie et al. catalogued the patent landscape for CRISPR technologies through 2014, finding hundreds of patent applications distributed across the globe. Since then, others have landscaped CRISPR patents in China, India, and South Africa, among other countries, and reached similar conclusions (Bire et al., 2021; Chowdhury and Gargate, 2021; Naidoo, 2020).

Meanwhile, the CRISPR technology itself has significantly evolved, beyond the Cas9 enzyme and basic forms of genomeediting, to synthetic forms of CRISPR enzymes and powerful, precise applications to make a variety of manipulations to the genome (Porto et al., 2020; Marzec et al., 2020). In addition, CRISPR has been used in ways other than basic genome-editing, including as a disease diagnostic, as a screening tool, and as a guard against deficiencies in other forms of CRISPR (Sanjana, 2017; Chertow, 2018; Zhang et al., 2017;, 2018). All of these variations and uses are likely patent protected in some fashion. A recent analysis by Martin-Laffon et al. (2019) found that 45% of CRISPR patents, worldwide were directed to technical improvements in the field, including the utilization of variants of Cas9, advances in sgRNAs their design, and “multiplexing,” making multiple edits simultaneously. At the same time, the reach of CRISPR patents is unevenly distributed by geography, with inventors from the United States and China being—far away—the leading applicants of CRISPR patents (Bire at al., 2021).

There is a strong expectation CRISPR technology will continue to be improved and continue to be patented. The academic literature demonstrates that CRISPR technology will continue evolve; new applications of CRISPR are announced frequently. As a consequence, there will be more patents covering various forms of CRISPR, held by many more players, in more than countries than current patent landscape analyses suggest. Much of this is a consequence of the technology’s susceptibility to “democratization,” i.e., its ability to be cheap, powerful, flexible, and easy to use (LaManna and Barrangou, 2018). At the same time, various forms of the technology are planned for large-scale commercial develop, which brings with it an increased risk of patent infringement lawsuits. These tensions illuminate how policies concerning CRISPR patents matter both more and less as governance instruments of the technology. While patents covering diverse forms of the technology are increasingly being held by a broader number of researchers and developers, their significance for commercial development means that they are increasingly likely to become arbiters of which variations are likely to be commercially development, who is involved in making such determinations, and how much such development costs. These considerations, in turn, intersect with the technology’s relationship with democratic power, institutions, and engagement and participation.

## DEMOCRACY AND GOVERNANCE OF CRISPR PATENT LICENSES

### Democratic Power and Public Interest

What is democratic governance? What do we mean when we say a government or system is democratic? Defining or identifying key markers of democracy help illuminate its qualities and evaluate how or why its absence is deleterious to society, to the public (as opposed to private) interest, or to general welfare. With respect to the patent licensing as an instrument of governance, two broader principles of democratic theory are worth exploring: The first is an understanding of democracy as an equalizing political power, redistributing power away from elites and toward a greater majority or group (Dahl, 1998). The second is a justification of this governance theory by analyzing its legitimacy (Buchanan, 2002). That is, is there intrinsic value in equitable distribution of political power or do democracies confer certain benefits or impose consequences on the public that makes democracy instrumentally desirable? When it comes to concepts such as equality, for example, many have argued that equality is either ontologically or instrumentally important, or perhaps both (Saffon and Urbinati, 2013). Patent licensing requires policymakers to choose—explicitly or not—whether they (and the polity) prefer outcomes in which benefits are distributed equally themselves or are concentrated only for some, by their users. This suggests an important opportunity and potential for deliberative engagement given that significant priority setting and value creation emerges in these governance choices.

In the case of CRISPR, one of the primary concerns is that its powerful effects will be used to benefit some groups disproportionately and create discriminatory outcomes for others. Patent licenses have the ability to maintain the high cost of therapies, to consequently affect the availability of insurance coverage, and to stymie competition. In addition, patent licenses often shape which diseases are studied for commercial development (and, therefore, which therapies are available to the public). That is, “it is precisely the novelty and power of CRISPR—and the potential effects of its patent landscape on the public health—that counsel us to solve these problems before it is too late for patients” (Sherkow, 2017). To be clear, this calculus is not different in kind from patent licenses for other therapeutic technologies. But CRISPR’s power to treat if not cure a great many unevenly distributed genetic diseases means that the distributional choices made by licensing governance is likely to have an outsized effect on the polity. Governing institutions should therefore be accountable to them. Choosing licensing regimes more (or less) responsive to public input is a choice tied up with varying theories of democratic control of—and equality of access to—novel technology.

Yet this is not to say that such choices should primarily focus on equal access as an end in itself. Given the nature of patent licensing—almost always with at least one private actor involved—these benefits should be primarily evaluated instrumentality. Do they get us what the public wants, even if achieved by private actors? Democracies are not only desirable descriptively, but legitimate insofar as a broader distribution of power creates the conditions for bettering public welfare (Anderson 2009). This may include the public choice to encourage the commercial development of CRISPR for some rarer diseases more so than common ones.

This may have implications for private licensing insofar as greater government reliance on private licensing to regulate technology means that the government becomes more and more accountable to private interests, and less so to the public’s interest. This is, in some ways, concerning with respect to ethical licensing restrictions as imposed by the Broad Insitute on its CRISPR patents. Relying too much on this model risks corrupting democratic institutions regarding technology distribution by eroding their ability to distribute resources equitably. In other words, efforts to correct such reliances after the technology has expanded becomes stymied as they become increasingly captured by private interests, giving sway to their desire to earn a profit or thrive above considerations of the public (Carpenter, 2014; Contreras and Sherkow, 2017).

To be clear, these aims are not always at odds. The development of COVID-19 vaccinations, for example, marries aspects of the public interest with government’s efforts to distribute novel technology, even while *global* distribution efforts have faltered and remain vastly unequal (Georgieva et al., 2021). And there are pathways in which this can become malicious, such as when government fails to curb the power of technology monopolies and they come to dictate our daily functioning (Kahn, 2017). By giving people greater voice, accountablility, and ability to shape the rules and laws that affect their lives, the polity is better able to advocate against domination and for access, whether it is one desirous of CRISPR therapy or one antagonistic to it (Rahman 2017). With respect to legitimacy, this means the ways in which licensing rules are justified or deemed legitimate depends on whether a particular democratic arrangement of people does well to protect the most vulnerable among us. Majority rule, often considered the default democratic decision-making method, has the capacity to suffer tyrannical tendencies when it comes to just distribution of resources or rights protections—patent licensing or otherwise (Ober 2008). Patent licensing regimes purely dominated by a majority gives the public ineffective recourse when circumstances change.

### Democratic Institutions and Mechanisms

Democracies, at their best, equalize political power or, at least, create more equitable systems of power distribution. These can be harnessed toward supporting minority groups, bolstering marginalized populations, and giving agency to those in other vulnerable conditions. But how should democracies arrange their institutions to fulfill these ends in practice? The main theory of democratic institutions borne out of the Enlightenment has been a representative system of governance, one in which we choose the people who choose the laws for us. Some of this justification has been practical: having everyone decide on everything—say, via plebiscite—can prove time consuming and overly laborious. The main substantive reasons though are rooted in a deep distrust of the larger polity to make decisions that foster their own self-determination (Sztompka, 1998). The fear of the unruly mob or the whims of the populous has sustained the ways in which democracies construct institutions around limiting the power of individuals or lay groups. This includes the United States Constitution, which has consistently incorporated a system of sepration of power among the three branches of government, including checks and balances, as a solution to “factionalism” (Federalist No. 10, 1788). Filtering the feelings and views of the public through their representatives has long been seen as an important way to contain the people’s unchecked “passions” (Holmes, 1995; Sabl, 2002) while also capturing their principal aims.

And so, too, with governing technology through patent licensing. The bulk of patent licensing regimes readily available are public in nature, with political representatives if not at the helm, accountable to their constituents. March-in rights, government use rights, and compulsory licenses are all effectuated by political actors who—historically at least—wield such power on behalf of (or at least with an ear toward) their constituents. Recent threats of government use rights, for example, were born from the wellspring of constituent demands (Brennan et al., 2016). And yet, not all constituent demands—even popular ones—have achieved changes in patent license objectives. The case of patented AIDS medicines in in the 1980s is notable (Grossman, 2016). So too, perhaps, are patent waivers—thus far not enacted—for COVID-19 therapies and vaccines (2 October Waiver Request, 2020; 25 May Waiver Request, 2021).

Within this context, therefore, it is notable that it is the legislature—rather than, say, an elitist judiciary—that has become the principal site of patent licensing policy and nuance. In the modern age, democratic rule typically means electoral democracy, with the mark of a “healthy” or “stable” democracy one in which there are frequent and fair elections of representatives (Urbinati,2006). “Mirrored representation”—where representatives who demographically mirror their constituents and can directly attest to their lived conditions —may even further such governance through patent licensing’s instrumental aims (Fishkin, 2013). This theory of a more direct or participatory democracy has particular implications for CRISPR patent licensing governance insofar as it traffics on the technology’s use to treat (or “cure”) certain forms of disability. Under this theory, the best representative to oversee genome editing patent licenses for a particular condition are those who suffer from the condition themselves. This allows these representatives the knowledge (and political cache) to determine how best to distribute genome editing technologies that can eliminate or modify certain genetic diseases, how such therapies get distributed, and who stands to benefit (or not) from certain forms of access. This removes these decisions from companies in charge of designing such therapies which, while knowledgable about the disease, are more likely moved by market research of demand and principles of profit maximization rather than balancing specific rights of access and advancing societal welfare.

The relationship between profit-maximizing capitalism and representative democracy is comple. While we do not pretend to fully untangle and resolve it here, we do note that patent governance has the potential to be responsive to such democratic interests or remove them from the public entirely. This is analogous, perhaps, to regulatory agencies mandate to protect the public from private interests’ cost-cutting, a bulwark protecting safe, reliable, and healthy products for consumption. Increasing the distance from the sight of decision-making runs the risk of making representative legislatures less democratically accountable even as it serves an important function in the system.

## DEMOCRATIC ENGAGEMENT AND PARTICIPATION

What role then do or should the populous have in engaging directly in democratic institutions? While there is good precedent in including people through deliberative opportunities in one-off events, what would democratic engagement in patent license governance look like if it were institutionalized? Does this governance construct even make sense given that most people may not even know what patents are, how CRISPR works, or what impacts this intersection may have on their lives?

There are roughly two ways of thinking about public engagement through deliberation: One is through creating or carving out systems of inclusion, such as town halls or participatory budgeting. The other is through allowing groups to participate by creating collective power that acts on institutions from the outside, such as patient advocacy groups that organize to pressure rightsholders to understand their views and push for resources (or object to such work). Beyond these formal collectives, there is renewed interest in creating randomly selected bodies of lay people, like a citizens jury, to assess public perceptions of new technologies (Burgess 2012). If given the right institutional space, resources, and tools—especially in a diverse and well moderated group—these “Citizen Assemblies” have yielded promising results in fostering people’s ability to understand and analyze complex problems of technical governance, to interact thoughtfully with one another, and arrive at rights-protecting collective judgments (Farrell, Harris, & Suiter 2019). Recently, they have been used in such places as Scotland, Ireland, France, and Belgium to understand what the people truly want and to experiment with a modified form of direct engagement (Carolan, 2015; McKerrell, 2019, Caluwaerts & Reuchamps, 2014; Fabre et al., 2021). Most recently and relevantly as well, a citizen jury was convened in Austrailia to weigh in human genome editing (“Australia Citizens Jury on Genome Editing, 2021). Conveners of the Australian Citizen Jury aim for a more global event convening paritcipants from around the world to deliberate together (Dryzek et al., 2020).

For genome editing technologies like CRISPR, deliberative forums are likely to include a variety of disability rights groups and environmental protection organizations who are certain to have, for some use cases, diverging views. A number of groups, for example, have advocated for patent holders to turn their attention to particular, oft-neglected diseases, including Duchenne muscular dystrophy (Miller, 2019). Some of this work is encouraged, if not mediated, by major CRISPR patent holders, including the University of California’s Innovative Genomics Insttute, 2021. Oppositely, an organized group of citizens in Key West, Florida repeatedly protested against the use of genomically edited mosquitos from being released into the environment, with some early success in halting field trials (Joseph, 2016). As with patented AIDS mediciations, this kind of power—at least in the United States—is a hallmark of democratic governance of technology. Akin to voting, these protections to petition one’s government creates conditions for lay people to organize with the goals of either endorsing or protesting technical developments of consequential import.

At the same time, few of these groups or their allies have considered ways in which patent licensing could effectuate their goals, either through representative government or directly, to patent holders. One notable exception is MIT professor Kevin Esvelt’s proposal to use patent licensing (and the threat of infringement) to police CRISPR “gene drive” technology—a strategy that has been noted by some advocacy groups (Guerrini et al., 2017). In this way, participatory democracy has an outlet to almost all of the patent licensing regimes above—both those where representative government is the medium through which licensing governance occurs, but also instances of “ethical licensing” by private entities. Further, such licensing governance operates at a scale appropriate and achievable for such groups—retail, condition-by-condition or gene-by-gene advocacy—rather than a wholesale restructuring of the country’s technology ecosystem more appropriate for expansive government intervention.

Given these conditions, political theorists interested in using patent licensing as a form of democratic technology governance should consider ways to reduce the democratic deficit of these spaces of power and decision-making authority. And in conjunction, those interested in patent governance should consider ways in which deliberative forums like mini-publics are being included in governance decisions around the world (Dryzek et al., 2020). Including the voice of the people in patent licensing decisions, both from represented stakeholder groups like the disability rights advocate organizations and in the form of random selection like Citizens Assemblies, can be of incredible importance for the public, affecting upstream moments of private and capital interests as well downstream impacts on the distribution of scarce resources and people’s lives. Forgoing such choices leaves participatory democracy to belatedly organize after significant decision making has already occurred.

These kinds of deliberative forums, also known as mini-publics, have varied in kinds, sizes, and selection mechanism and could be convened by institutions responsible for patent licensing decisions, including private entities or, in the case of public licensing, Congress. Similarly, the question of patent governance could included on the agenda of broader deliberative events about technology governance, such as the Australian Citizens Jury. While most mini-publics currently play an advisory role, it is conceivable that, as they gain traction, their instrumental value in risk-governance and modes of engagement becomes more politically acceptable. If licensors grant them increasing levels of authority, they stand to actually impact the law.

Whereas many people consider the lay public less capable of reaching certain decisions on such technical issues, evidence from such mini-publics as Deliberative Polls and other assemblies shows that mini-publics actually have good capacity for learning and understanding technical material, and weighing risk in ways that are helpful value-based perspectives (Fishkin 2019). Given that groups of experts and elites themselves suffer from certain problems of exclusion, such as silo effects and skewed risk assessments, combining experts’ work with those from deliberative forums creates the opportunity for greater diversity having in risk assessment and weighing private v. public interests (Scheinerman 2019).

## CONCLUSION

Once the province of the arcane, patents should become a larger part of the conversation concerning technology governance, like that surrounding genome editing, for they are a powerful form of technology governance. Licenses, that is, permissions to use patented technologies, determine who, what extent, and under what terms others than can use them. These include a variety of government set licensing regimes that do just that, including licensing regimes sought by the government itself, march-in rights, government-use rights for others’ patented technologies, compulsory licenses, and restrictions on licenses, to say nothing of private governance regimes with ethical limitations. These licenses are themselves a form of democratic oversight that gives the public the capacity to preventing purely private interests from superseding their own, especially in ways that are dominating, oppressing, or otherwise harmful or unjust.

These patent licensing regimes also often intersect and have different democratic purchase. Some, like march-in rights, are effectuated only through the filter of representative democracy, and even then are rarely, if ever, acted upon. Others are subject to non or even antidemocratic norms in a variety of exclusionary ways. At the same time, participatory democracy has the potential to shape patent licensing regimes according to popular will (or whim), an effort to control the development and distribution of technology in ways small enough to be effective, both at the level of government, down to individual patent holders.

This a critically important mechanism for a technology like CRISPR, one with such heated public interest and with such intense calls for public accountability. The technology’s applications have grown tremendously alongside increased calls for public engagement. There are serious concerns that certain forms of technology, as released to the wider public, will be permanent facets of society foisted on it without its deliberative input. Meanwhile, the CRISPR patent estates, although once held in the hands of few, are rapidly expanding.

Facilitating and incorporating public input for such an expansive technology will likely be a long and difficult task. It is further unclear what, exactly, the public wants for such a wide-ranging technology that has so captured its imagination. Previous efforts, like public commissions, community representatives on government panels, may not be successful to garner a definitive view. Smaller, piecemeal efforts at public engagement over licensing regimes, both public and private, may contribute to better digestible—and more effective—forms of democratic technology governance both because they can be asked a more targeted policy question and because they are situated upstream of further distribution policy questions. Using the patents allows the public to better control technology under currently established legal regimes and do so in a way the public deems equitable. The public is better armed to mitigate the domination of private interests.

Whether this is viable remains to be seen. Scholars should look to see whether patent licensing regimes are, in fact, being used by government to control genome editing technology and whether the public, through the procedures of deliberative or participatory democracy or otherwise, is interested in using patent restrictions as a governance mechanism. Scholars may also further examine licensing restrictions to see what the terms are and how they are generated.

The power of CRISPR, as a technology, ultimately has strongly democratic features insofar as it is the most equitably distributed gene editing tool. It can be used by almost anyone, anywhere in the world, with minimal training and inexpensive reagents. Yet, the technology’s commercial development—and some of its most egregious applications, real and, to date, fictional—have sailed over many democractic controls otherwise taken for granted. Democracy, like CRISPR, can be a powerful corrective technology for the ills of society.

## Footnotes

1 Although we focus on licensing in this paper, it is also the case that patentability criteria—that is rules regarding which technologies can be patents—similarly involve choices about democracy and polity. That, of course, is a complicated mixture of a variety of branches of government, some of which have more democratic features or procedures than others. For more on this point, see Part B.
